# Remarkable Instance of Diseased Bladder

**Published:** 1826-07

**Authors:** 


13. *Remarkable Disease of the Bladder.*† A youth, aged 18 years, had enjoyed excellent health till the autumn of 1822, at which time he was seized suddenly with incontinence of urine, without any ostensible cause. This symptom disappeared in a few days, leaving much pain in the region of the bladder, with a constant desire to make water. In a short time after this, complete ischuria took place, but soon went off, after the discharge of a fleshy substance *per urethram*. A temporary respite from suffering succeeded this last event; but other morbid phenomena soon took place, and the patient was worn out by a series of tormenting afflictions.

*Dissection.* A great quantity of purulent matter was found in the cavity of the abdomen, and an encephaloid mass, the size of a pigeon's egg, adhering, on one side, to the pubis, and on the other, to the bladder, the coats of which were converted into a similar morbid growth, with numerous small fungous bodies on its interior surface. The coats of the bladder generally were thickened, and the orifice of one of the ureters obliterated. That ureter was distended to the size of the intestinal canal.

† Dr. Paderborn. Rheno-Westphalian Annals of Medicine and Surgery, 1825.

